# Lessons Learned for the National Children’s Study from the National Institute of Environmental Health Sciences/U.S. Environmental Protection Agency Centers for Children’s Environmental Health and Disease Prevention Research

**DOI:** 10.1289/ehp.7669

**Published:** 2005-06-24

**Authors:** Carole A. Kimmel, Gwen W. Collman, Nigel Fields, Brenda Eskenazi

**Affiliations:** 1 National Children’s Study Interagency Coordinating Committee, National Center for Environmental Assessment, Office of Research and Development, U.S. Environmental Protection Agency, Washington, DC, USA; 2 Division of Extramural Research and Training, National Institute of Environmental Health Sciences, National Institutes of Health, Department of Health and Human Services, Research Triangle Park, North Carolina, USA; 3 National Center for Environmental Research, Office of Research and Development, U.S. Environmental Protection Agency, Washington, DC, USA; 4 Center for Children’s Environmental Health Research, School of Public Health, University of California, Berkeley, California, USA

**Keywords:** asthma, autism, children, environmental health, National Children’s Study, NIEHS/EPA Children’s Centers, obesity, pregnancy

## Abstract

This mini-monograph was developed to highlight the experiences of the National Institute of Environmental Health Sciences (NIEHS)/U.S. Environmental Protection Agency (EPA) Centers for Children’s Environmental Health and Disease Prevention Research, focusing particularly on several areas of interest for the National Children’s Study. These include general methodologic issues for conducting longitudinal birth cohort studies and community-based participatory research and for measuring air pollution exposures, pesticide exposures, asthma, and neuro-behavioral toxicity. Rather than a detailed description of the studies in each of the centers, this series of articles is intended to provide information on the practicalities of conducting such intensive studies and the lessons learned. This explication of lessons learned provides an outstanding opportunity for the planners of the National Children’s Study to draw on past experiences that provide information on what has and has not worked when studying diverse multiracial and multi-ethnic groups of children with unique urban and rural exposures. The Children’s Centers have addressed and overcome many hurdles in their efforts to understand the link between environmental exposures and health outcomes as well as interactions between exposures and a variety of social and cultural factors. Some of the major lessons learned include the critical importance of long-term studies for assessing the full range of developmental consequences of environmental exposures, recognition of the unique challenges presented at different life stages for both outcome and exposure measurement, and the importance of ethical issues that must be dealt with in a changing medical and legal environment. It is hoped that these articles will be of value to others who are embarking on studies of children’s environmental health.

The series of six articles in this mini-monograph developed out of a desire to learn from the collective experiences of the Centers for Children’s Environmental Health and Disease Prevention Research (hereafter Children’s Centers) in a way that could be useful for the design and implementation of the National Children’s Study. The Children’s Centers are co-sponsored by the National Institute for Environmental Health Sciences (NIEHS) and U.S. Environmental Protection Agency (EPA) and conduct both observational studies of etiology and intervention studies. Several of the exposures and outcomes studied by the Children’s Centers are of interest for the National Children’s Study. This mini-monograph highlights the experiences of the Children’s Centers’ studies, and the articles are meant to serve as a primer of lessons learned for the National Children’s Study. The articles represent a synthesis of thoughts on several topics: methodologic issues for conducting longitudinal birth cohort studies (Eskenazi et al. 2005) and community-based participatory research (Israel et al. 2005) and issues in the measurement of air pollution (Gilliland et al. 2005), pesticide exposures (Fenske et al. 2005), asthma (Eggleston et al. 2005), and neurobehavioral toxicity (Dietrich et al. 2005). Summarizing the combined experiences of the Children’s Centers at this time afforded an opportunity to inform the planning and protocol development of the National Children’s Study and to provide information on what has worked and what has not worked when studying diverse multi-racial and multiethnic groups of children with unique urban and rural exposures.

## The National Children’s Study

The idea for the National Children’s Study originated from the Developmental Disorders Workgroup of the President’s Task Force on Environmental Health Risks and Safety Risks to Children, established in 1997 as a result of Federal Executive Order 13045, signed by President Clinton on 21 April 1997. This Executive Order, titled “Protection of Children from Environmental Health Risks and Safety Risks” (Clinton 1997) required federal agencies involved in related activities to consider special environmental circumstances that could pose a health threat to children. In late 1999, the task force approved the exploration of the feasibility of the Longitudinal Cohort Study of Environmental Effects on Parents and Children (later renamed the National Children’s Study). Subsequently, the Children’s Health Act (2000), which Congress passed unanimously in October 2000, authorized the planning and implementation of this study. The Children’s Health Act charged the director of the National Institute of Child Health and Human Development/National Institutes of Health together with a consortium of federal agencies, including the U.S. EPA, the Centers for Disease Control and Prevention, and other appropriate agencies to plan and implement this national longitudinal study of environmental influences on children’s health and development. The NIEHS shortly thereafter became the fourth lead partner agency. The Children’s Health Act broadly defined the environment to include physical, chemical, biologic, and psychosocial factors. Further, the act called for investigation of basic mechanisms of developmental disorders and environmental factors, both health adverse and health promoting.

Plans for the National Children’s Study are to enroll pregnant women as early in pregnancy as possible and to enroll a subset of the cohort before conception. Current plans are to follow 100,000 births to adulthood (21 years of age), with collection of data on exposures and outcomes at points throughout the study. Two major goals of the study are to use the longitudinal design as a way to link exposures with outcomes that occur at different points in time (i.e., exposure may precede the resulting outcome by months or years) and to explore interactions among various factors, including genetic traits. The general design and administrative structure are described by the National Children’s Study Interagency Coordinating Committee (2003) and the Study Plan (National Children’s Study 2005).

Planning for this large study began in mid-2000 with the initiation of a number of methods development studies, particularly the development of less burdensome and less costly exposure assessment methods (U.S. EPA 2004a). Other efforts have included laboratory studies on noninvasive procedures for analysis of biomarkers of exposures and outcomes (Rockett et al. 2002), development of comparable measures that could be administered in children and laboratory animal models enabling further exploration of exposure–outcome links in animal studies (Sharbaugh et al. 2003), and field studies using focus groups to explore recruitment and retention issues. A number of reviews and white papers have been developed, including an evaluation of advanced technology for capturing data, a review of the leading hypotheses of the effects of environmental factors on children’s health outcomes, and a review of the leading sampling strategies for consideration in the study. In addition, a resource database on biomarkers of exposures and outcomes was developed (U.S. EPA 2004b). More recently, a number of workshops have been organized to explore and refine the measures that might be included in the protocol for the study. A complete listing of workshops, meetings, and reports can be found at the National Children’s Study website (2005).

Because of the research being conducted by the Children’s Centers, a good deal of expertise and specialized knowledge have already been developed that can be particularly valuable to the development of protocols for the National Children’s Study. As detailed below, much of the work of the original eight centers was on pesticide exposures and neurodevelopment, and air pollution and asthma. The four additional Children’s Centers are focusing on developmental disabilities, particularly autism spectrum disorders, and the impact of various environmental pollutants on learning and behavior. These and other exposures and outcomes have been established as priorities for the National Children’s Study (Table 1). Thus, the Children’s Centers have experience in several of the priority focus areas that should provide valuable input to the design of the National Children’s Study.

In addition to these priority focus areas, it is recognized that community participation and involvement are key to the success of the National Children’s Study. The study design is likely not to be strictly community based, but rather will involve multiple sites throughout the country with a complex multilevel sampling strategy for obtaining the most representative population possible for inclusion in the study. Perhaps the greatest lesson to be learned from the Children’s Centers is the myriad of logistical complexities to be considered in an intensive investigation of children’s exposure and health outcomes. The cumulative experience of the Children’s Centers in conducting community-based participatory research, particularly in underserved populations, will provide valuable information for future studies.

## The NIEHS/U.S. EPA Centers for Children’s Environmental Health and Disease Prevention Research

The same federal executive order that spawned the National Children’s Study also led to development of the Children’s Centers. The NIEHS and U.S. EPA collaborated in 1998 to develop a research program that would bring together efforts to better understand the exposures of infants and young children, study the health effects of such exposures to better understand the mechanisms by which they work, and explore intervention strategies for reducing such exposures in a way that would provide evidence for practice. The program was funded in two phases; the first phase in 1998 funded eight centers, and four more centers were funded in 2001. In 2003, a new round of competition was completed and seven awards were made. Six of the pre-existing centers continue to date, and a new center was added. The motivation for this program and a summary of the first eight centers has been more fully discussed by Dearry et al. (1999) and O’Fallon et al. (2000). A full description of the Children’s Centers can be found on the NIEHS website (NIEHS 2005).

The purpose of the Children’s Centers program is 2-fold: first, to create local research environments that promote multidisciplinary interactions among basic, clinical, and behavioral scientists through university/community partnering in order to accelerate translation of basic research findings into clinical prevention or intervention strategies; and second, to support a coordinated nationwide network of scientists and community advocacy groups synergistically sharing their experiences to address relevant questions related to the role of environmental exposures in the health of children in order to enhance community-level capacity to identify and address environmental threats and prevention opportunities. The aims of establishing this national network are to foster communication, innovation, and excellence in children’s environmental health; to provide training opportunities for scientists and clinicians for future development of this field of study; and to broaden the national discussions between diverse groups of community advocates and organizers on common interests in protecting and nurturing healthy environments for children.

Each center is designed around a central theme focusing on important questions in understanding the role of exposures in one of the following health outcome areas: respiratory disease, childhood learning, and growth and development including developmental disabilities. Exposures to toxicants such as polychlorinated biphenyls (PCBs), mercury, lead, air pollution, allergens, agricultural and urban pesticides, second-hand smoke, and others are part of the Centers Program’s research priorities. All centers have multiple projects across scientific disciplines, from basic laboratory-based research and genetics to exposure assessment, epidemiology, and clinical trials. Methods have been developed, field tested, and implemented to detect and define health symptoms and outcomes; new exposure technology has been developed to assess environmental exposures and body burden in a diverse array of biospecimens; and creative approaches to reaching and retaining traditionally “hard to follow” socioeconomic groups have been implemented with community input.

Over the past 5 years and using a variety of methods, scientists from the NIEHS/U.S. EPA Children’s Centers Program have made a number of advances that would not have been possible without the establishment of a coordinated network of centers to foster multi- and interdisciplinary research targeted at understanding children’s environmental health risks and reducing them. Many of the results of these collaborations and interactions have implications for children’s environmental health and the National Children’s Study. For example, studies have shown that blood and urine specimens from pregnant women show measurable levels of pesticides, suggesting that the fetus is exposed to these chemicals during early development (Bradman et al. 2003; Whyatt et al. 2003); children in urban and rural environments are exposed to a complex mix of agricultural and household pesticides, environmental tobacco smoke, and polycyclic aromatic hydrocarbons and negative social factors that can affect their early growth and development (Berkowitz et al. 2004; Eskenazi et al. 2004; Perera et al. 2004; Rauh et al. 2004); and exposures to lead in the urban environment can have lifelong effects such as behavioral problems and criminal behavior in early adult life (Ris et al. 2004). Research on air pollution and asthma has broadened our understanding of the inflammatory process in the lung (Walters et al. 2002) such that the effects of air pollution can be seen in school-age children as increased exacerbation of asthma symptoms and increased days absent from school (Gilliland et al. 2003; McConnell et al. 2003). Intervention studies have been conducted to show that the household environment can be cleaned up in a way to significantly reduce allergens from dust mites and cockroaches that should reduce the incidence of asthma symptoms in children (Eggleston et al. 2004).

### Community-based participatory research.

A requirement for every Children’s Center is the inclusion of one project that uses community-based participatory research methods. This type of methodology encourages full participation of the community in the design, implementation, evaluation, and translation of the research. Research ideas may begin with the concerns of the community. Community partners, scientists, and clinicians share knowledge of exposure, health effects, and prevention strategies. Many studies train and employ community members as study coordinators, interviewers, or environmental technicians. Community participation ensures the relevance of the research questions and appropriateness of the research strategies. Research results are disseminated back to the community on an ongoing basis through community advisory boards, newsletters, health fairs, and other educational activities.

### Cohorts and study designs.

Most of the centers have established cohorts of children in which to study the dynamic relationship between exposures to environmental agents and health outcomes. In general, two distinct age groups are targeted: birth cohorts where pregnant women were enrolled and their offspring become study participants, and cohorts of school-age children enrolled either in school settings or in a medical care environment. The birth cohort studies seek to understand exposures during fetal development and health risks related to respiratory illness progression and neurodevelopmental effects, including motor, sensory, and cognitive deficits. Because asthma cannot be definitively diagnosed until ages 3–4, prospective follow-up of a group of young children provides for new opportunities as they age. The school-age cohort studies focus on asthma, and children are recruited through school classrooms, neighborhood health clinics, and other medical care settings. One large cohort study of school children in Los Angeles, California, that was started 10 years ago continues to follow the children and compare genetic factors from recently collected specimens with historical air pollution and medical data. Case-only or case–control designs are used in two other studies of children that focus on understanding the possible environmental causes of autism, a relatively rare disorder. Intervention/prevention studies include cohorts of children with disease or unique exposures that can be found in urban and rural settings. These studies are unique with regard to community participation, recruitment and retention, and dissemination of study results; Table 2 lists the types of studies and centers.

### Government/Children’s Centers partnerships.

The NIEHS and U.S. EPA, as federal funding partners, continue to align their priorities and working relationships to manage and support this $140 million program. The agencies’ commitment to overcoming differences in their regulatory and research mandates is reflected in the broad success of the Centers Program’s impact on public policy and influence in several fields of public health. The federal partners share responsibility in both supporting the national network of researchers and sponsoring annual center meetings. Bringing center scientists and community members together on a consistent basis has been instrumental in the success of these programs. This created a stronger working relationship across the Children’s Centers than would have been fostered with individual programs working alone. At the inception of the program, many meetings were held to discuss definitions of health outcomes and ways to measure them, methodologies for exposure assessment, questionnaire items, and follow-up strategies with special attention to retention of study participants, cultural sensitivities, and engagement of community. Information was shared and protocols were designed for individual studies that strove for commonality. The goal was not to have standardized methods employed, but rather to see where collaborations could be built and methodologies shared.

The NIEHS/U.S. EPA Children’s Centers Program is now in its seventh year. The program has generated important scientific results and expanded our knowledge of exposures to young children and how they affect their health status. There is a wealth of knowledge about issues that pertain to conducting future studies in this field, especially the National Children’s Study. This mini-monograph is an attempt to describe in detail the lessons learned from these important groundbreaking studies.

## Major Lessons Considered Important for Planning the National Children’s Study

Several major lessons from the Children’s Centers are important for consideration in planning the National Children’s Study. These and a number of others are discussed in detail in the articles in this mini-monograph (Dietrich et al. 2005; Eggleston et al. 2005; Eskenazi et al. 2005; Fenske et al. 2005; Gilliland et al. 2005; Israel et al. 2005).

First, long-term studies that follow participants into adolescence and early adulthood are considered essential to assess the full range of developmental consequences of exposure to environmental chemicals.

It is also important to identify a population with a wide range of exposure concentrations for those key pollutants hypothesized *a priori* to be of interest in order to evaluate the relationships between the distributions of multiple exposures and observed effects.

It is necessary to allow for population differences in literacy, language, and culture when establishing study procedures for recruitment and retention and in determining the type of information collected and the methods of collection.

Assessment tools need to balance measures both broad and narrow in scope. Questionnaires, neurodevelopmental instruments, and the like employed in these studies should include a core set to evaluate the entire cohort and additional segments for selected populations that may be unique based on their exposure or other attributes.

Exposure assessment should include a combination of environmental and personal measurements as well as data derived from questionnaires and from observational and ecologic data. The exposure assessment effort should take advantage of modeling approaches to provide estimates for the entire cohort. Targeted exposure studies in a selected sub-sample of study subjects may be useful for improving exposure assessment. The depth of assessments that can be realistically implemented will be restricted in populations that are widely dispersed geographically, have limited transportation, or lack trained personnel in the community.

Procedures for monitoring the quality and accuracy of data collection must be established and maintained not only for the collection and analysis of biologic or environmental specimens, but also for the assessment of questionnaire, developmental testing, and other health outcome data. Data safety and monitoring procedures must be in place.

Active and meaningful participation of the community is essential for determining the relevant research questions, enrolling and retaining the cohort in an intensive investigation over the long term, and contributing to translation of scientific principles and research results for communities and the public at large. This requires establishing trust and respecting differences in culture and knowledge of the community. Sufficient time and resources are necessary to develop community partnerships.

The ethical issues in a longitudinal birth cohort study are likely to become increasingly more complex in the changing medical and legal environment and must be carefully considered in designing research protocols and following the cohort. It is necessary to develop clear plans of referral when children with disease, developmental difficulties, or adverse social situations emerge.

Communication of risk to participants and the community and translation of research findings into interventions and policies are of utmost importance and should be part of the research plan and cost consideration.

Funding for a longitudinal birth cohort study must be adequate for the start-up period, continuous without gaps, and long term. Costs have often been underestimated because tracking and maintaining study participants is labor intensive.

The unique characteristics at each developmental stage from birth through adulthood must be considered. Every age presents special challenges in both outcome and exposure assessment.

Finally, the health and development of children are multifactorially determined. The greatest challenge is anticipating the data and specimens that will allow the questions of the future to be answered. This requires state-of-the-art tools for data collection and tracking participants, environmental and biologic specimen repositories, and anticipation of future human subject requirements in consent procedures.

The unique challenges faced by the Children’s Centers in studying diverse populations will be especially helpful for the National Children’s Study, which is intended to be a nationwide study representative of the many populations across the United States. Although the Children’s Centers have reported important findings from their individual studies, it is only by examining the collective experiences of the Children’s Centers in these lessons learned articles that we gain a better perspective of the potential challenges to be met in the many National Children’s Study sites.

## Figures and Tables

**Table 1:** Priority health outcomes and exposure areas identified for the National Children’s Study.

Priority health and disease outcomes
Pregnancy outcomes
Neurodevelopment and behavior
Childhood injury
Asthma
Obesity and physical development
Priority environmental exposures and other factors
Physical exposures and environment
Chemical exposures
Biologic environment and genetics
Psychosocial environment and exposures

**Table 2:** Primary outcomes, exposures, and populations studied by the Centers for Children’s Environmental Health and Disease Prevention Research.

Study	Outcomes or health concern	Exposures or intervention	Population
Birth cohorts
University of California, Berkeley	Infant growth and development	Pesticides, allergens	Latinos, in an agricultural community, Salinas Valley, California
Columbia University	Infant growth and development, asthma	Pesticides, lead, smoke, PAHs	Urban Latinos (Dominican), African Americans, NYC
Mt. Sinai Medical Center	Infant growth and development, obesity	Chlorpyrifos, endocrine-disrupting chemicals, built environment	Urban Latinos, African Americans, East Harlem, NYC
University of Illinois	Infant growth and development	PCBs, mercury	Hmong, Wisconsin
Cincinnati Children’s Hospital	Infant growth and development	Lead, pesticides, smoke	Urban African Americans, Cincinnati, Ohio
School-age cohorts
Johns Hopkins University	Asthma	Allergens, air pollution	African Americans, health care based, Baltimore, Maryland
University of Southern California	Asthma	Air pollution	School children in 12 communities in Los Angeles, California
University of Michigan	Asthma	Air pollution, allergens	African Americans, school based, Detroit, MI
University of Iowa	Asthma	RSV, endotoxin	Rural Iowa
Case-only and case–control studies
University of California, Davis	Autism	Environmental and other risk factors	Cases and controls identified throughout California, mentally retarded controls and healthy controls
University of Medicine and Dentistry of New Jersey	Autism, regression	Home chemical exposures	Autistic children with and without regression
Intervention/prevention studies
University of California, Berkeley	Agricultural pesticide exposure	Worker cleanup in fields	Migrant workers in Salinas Valley, California
Columbia University	Household pesticides and allergens	Integrated pest management	African Americans and Latinos, North Manhattan, NYC
Mount Sinai Medical Center	Chlorpyrifos use, cockroach allergen	Integrated pest management	African Americans and Latinos, East Harlem, NYC
University of Illinois	Birth outcomes and growth	Education about fish consumption (PCBs, mercury)	Hmong, Wisconsin
Cincinnati Children’s Hospital	Lead	Household cleanup and remediation	African Americans, inner-city Cincinnati, Ohio
University of Michigan	Asthma symptoms	Household cleanup	African Americans and Latinos, Detroit, Michigan
Johns Hopkins University	Asthma symptoms	Household cleanup	African Americans, Baltimore, Maryland
University of Iowa	Asthma symptoms	Cleanup, medical management, personalized care plan	Rural Iowa
University of Southern California	Asthma symptoms	Household cleanup	School children in 12 communities in Los Angeles, California
University of Washington	Agricultural pesticide exposure	Pesticide reduction strategies	Agricultural workers, Yakima Valley, Washington

Abbreviations: NYC, New York City; PAHs, polyaromatic hydrocarbons; RSV, respiratory syncytial virus.
